# Supplementary figures and images for: Neuronal mTORC1 inhibition promotes longevity without suppressing anabolic growth and reproduction in C. elegans
Source: PLoS Genet. 2023 Sep 18;19(9):e1010938. doi: 10.1371/journal.pgen.1010938 (PMC10538657; doi:10.1371/journal.pgen.1010938)

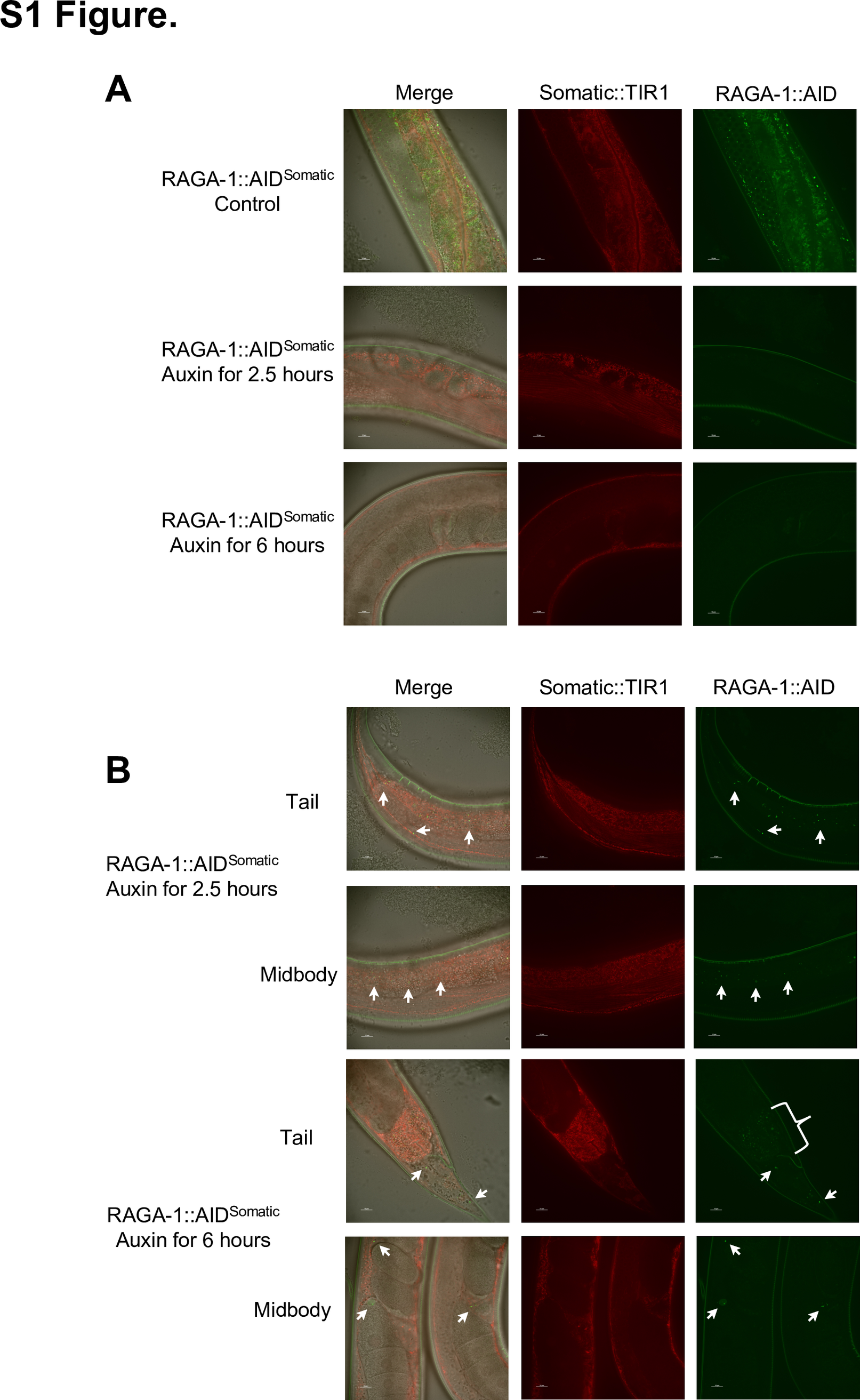

Supplement: S1 Fig — (A) AID of RAGA-1 in the midbody region. (B) Representative images of the worms with partial degradation of RAGA-1::AID (containing a few remaining puncta) at the corresponding time points. 2.5 hours after auxin treatment, RAGA-1::AID::EmGFP signal is diminished, but 7 out of 21 imaged worms still had between 1 and 20 green puncta. After 6 hours on 0.15 mM auxin, RAGA-1::AID is much more thoroughly degraded with only 6 out of 20 imaged animals still containing 1 or 2 GFP puncta. n = 2, at least 20 worms imaged per condition. White bracket indicates intestinal autofluorescence. Picture in last row shows two worms side-by-side. (TIF) [file pgen.1010938.s001.tif]

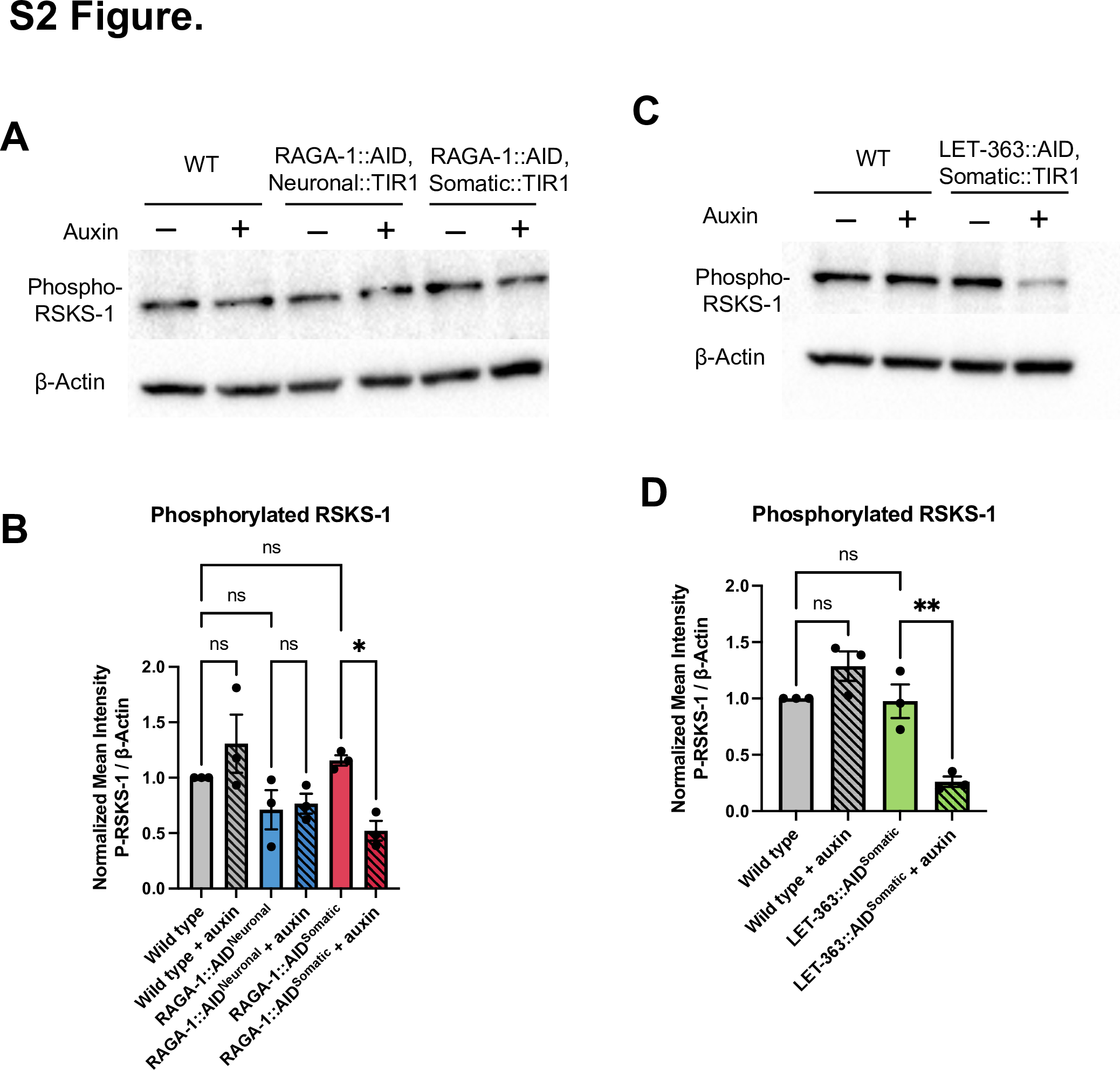

Supplement: S2 Fig — (A) Representative western blot of one replicate of P-RSKS-1 levels after AID of RAGA-1. (B) Quantification of all 3 repeats of the western blot indicated in (A). Animals were grown on control or 0.15 mM auxin plates from hatch and harvested at day 1 of adulthood. P-RSKS-1 levels are unaffected by auxin in wild type animals. RAGA-1::AIDNeuronal animals have no significant changes in P-RSKS-1 levels at baseline or upon auxin treatment. P-RSKS-1 is unchanged at baseline in RAGA-1::AIDSomatic animals but is significantly decreased in auxin-treated animals. (C) Representative western blot of one replicate of P-RSKS-1 levels after AID of LET-363. (D) Quantification of all 3 repeats of the western blot indicated in (C). Animals were grown on control plates form hatch, placed on auxin or control plates at day 1 of adulthood, then harvested on day 3 of adulthood. P-RSKS-1 levels are unaffected in wild type animals. In LET-363::AIDSomatic animals, adult-onset auxin-treatment significantly decreases P-RSKS-1 levels. For each replicate, intensity was normalized to the signal in wild type animals. Error bars are plotted at mean ± SEM. * indicates p < 0.05, ** indicates p < 0.01. (TIF) [file pgen.1010938.s002.tif]

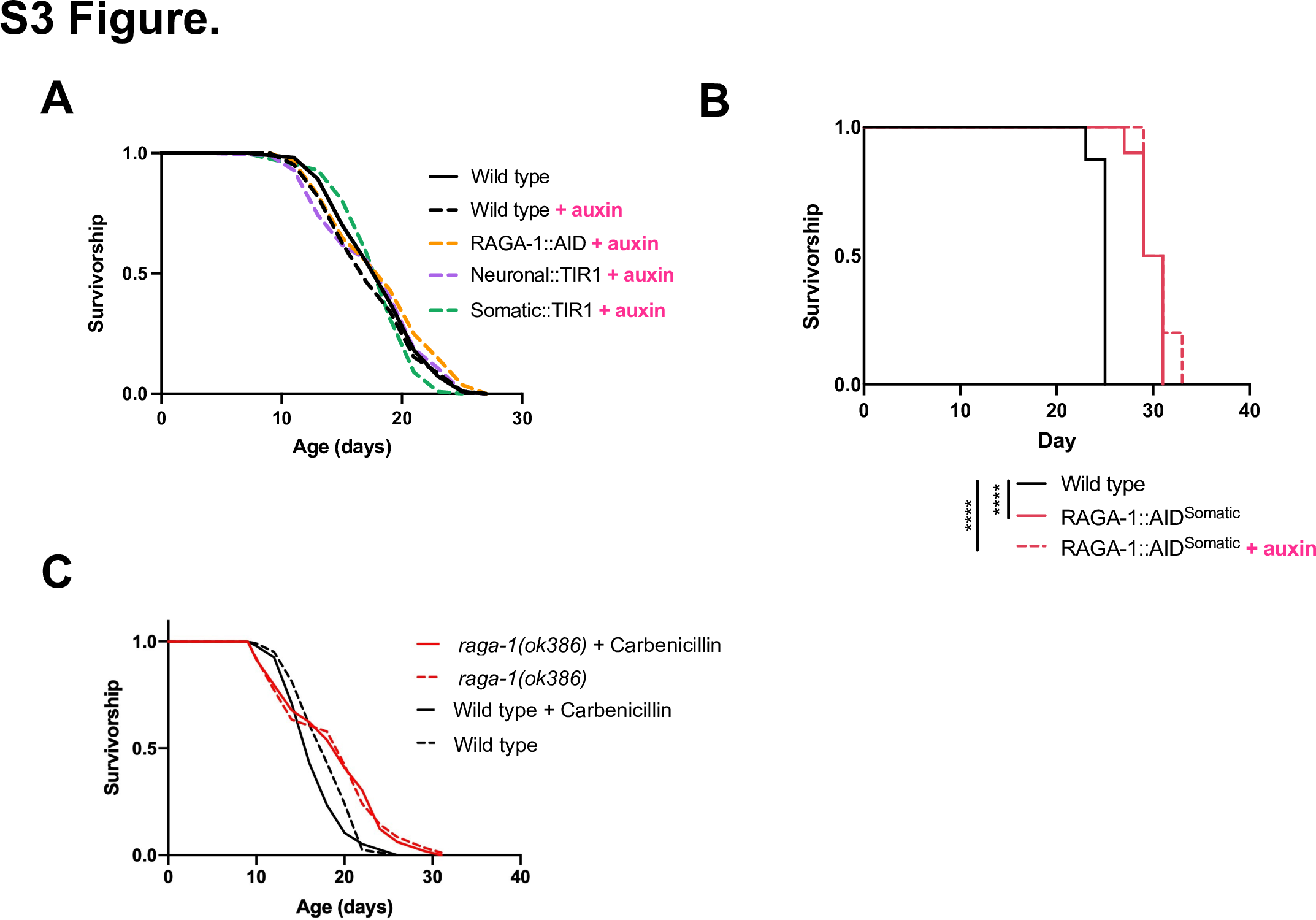

Supplement: S3 Fig — (A) Lifespan of RAGA-1::AID and TIR1 strains. Administration of auxin to wild type worms or worms containing AID-tagged RAGA-1, Somatic::TIR1, or Neuronal::TIR1 alone does not alter their lifespan (p = 0.4515, 0.279, 0.2494 and 0.9632 respectively). n = 3. (B) Maximum lifespan, plotted as the survival of the top 10th percentile of the population, is significantly extended for RAGA-1::AIDSomatic in both the absence and presence of auxin as compared to wild type animals (p < 0.0001 for both). (C) The survival curve for raga-1(ok386) mutants sometimes contains a “crash” in which the raga-1 mutants die even faster than wild type animals early on even though the latter portion of the population is long-lived. We have observed this phenotype in the presence and absence of antibiotics. (TIF) [file pgen.1010938.s003.tif]

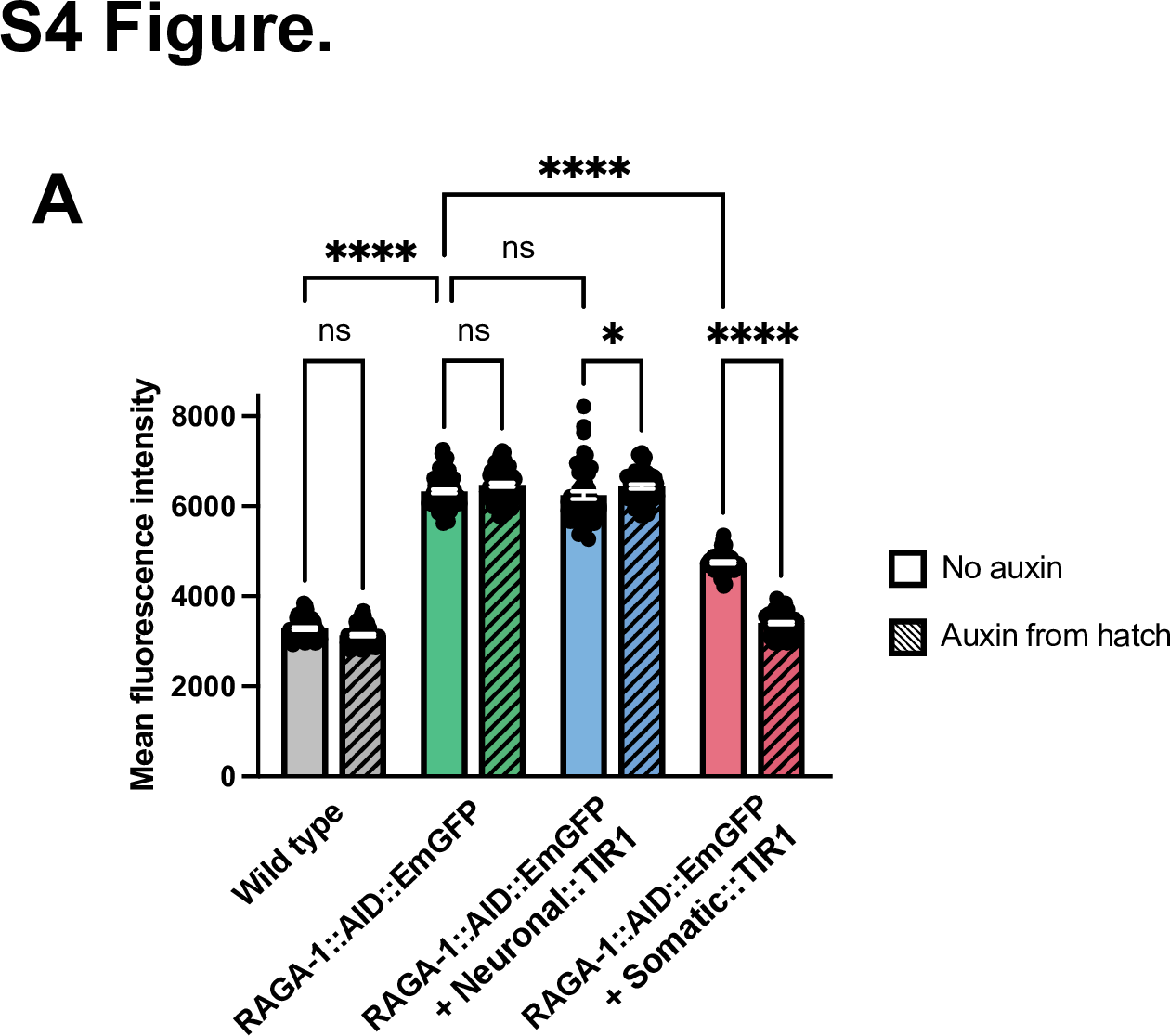

Supplement: S4 Fig — (A) Animals were grown on control or 0.15 mM auxin plates from hatch then imaged on day 1 of adulthood. Wild type animals have a baseline level of GFP fluorescence largely originating from intestinal autofluorescence. RAGA-1::AID::EmGFP animals without any TIR1 in the background have a higher GFP intensity than wild type animals, as expected, which is unaffected by auxin treatment. The addition of Neuronal::TIR1 doesn’t alter GFP levels. In contrast, the addition of Somatic::TIR1, even in the absence of auxin, significantly decreases GFP fluorescence, indicating auxin-independent degradation of the tagged RAGA-1. The addition of auxin further decreases GFP fluorescence in these animals close to the autofluorescence levels noted in wild type animals. Data points indicate the mean fluorescence for an individual animal. All animals imaged across 3 individual experiments are plotted together in this graph. Error bars are plotted at mean ± SEM. * indicates p < 0.05 and **** indicates p < 0.0001. (TIF) [file pgen.1010938.s004.tif]

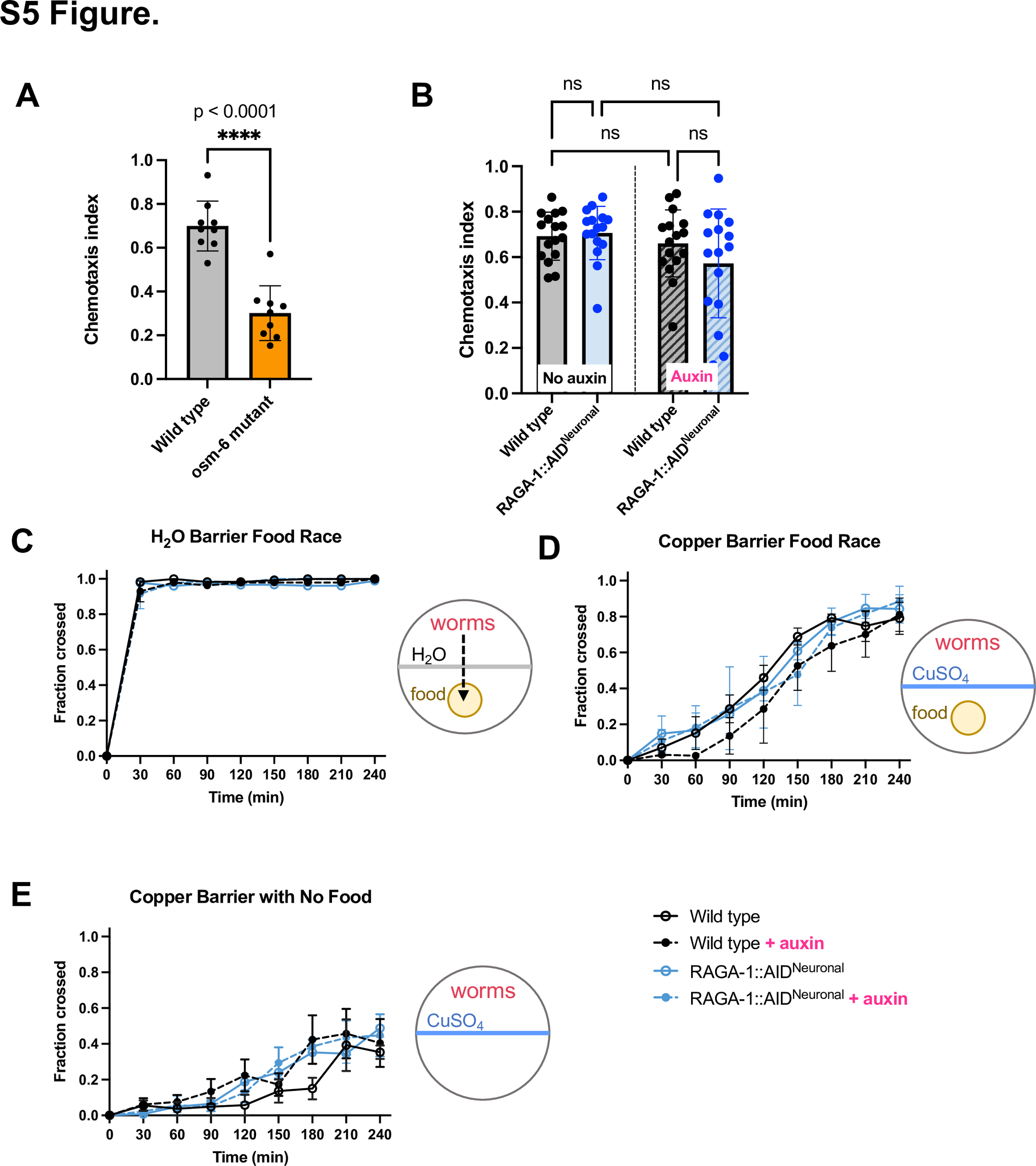

Supplement: S5 Fig — (A) Diacetyl attraction assay for wild type worms (N2) versus the osm-6 mutant (PR811) with impaired sensory neuron function. osm-6 mutants have a significant deficit in the ability to sense diacetyl. n = 3 independent experiments with 3 replicates per experiment. (B) Neuronal AID of RAGA-1 has no effect on C. elegans’ ability to sense diacetyl (p > 0.05 for all pairwise comparisons). n = 5 independent experiments with 2 to 3 replicate plates per experiment. Data was analyzed with a one-way ANOVA and, for (B), followed by multiple comparisons. (C-E) Copper barrier starvation assay. (C) Animals placed on the side of a plate with no food quickly cross the non-aversive water barrier to get to the side of the plate with food and remain there for the duration of the assay. Neuronal degradation of RAGA-1 doesn’t impair this food-sensing phenotype. (D) When placed on a plate with an aversive copper barrier in the middle and a food lawn on the other side, worms will, over time, cross the copper barrier to reach the food. Eventually, almost all animals will have crossed and be located on the food side. C. elegans with neuronal loss of RAGA-1 behave the same as wild type worms. (E) When placed on a plate that has no food on either side and an aversive copper barrier in the middle, C. elegans will, over time, cross the barrier in search of food so that any given time point some, but not all, of the animals will be over the barrier. Neuronal AID of RAGA-1 has no effect on this searching phenotype. For C-E, the behavior of the strains was analyzed with a two-way ANOVA with repeated measures. No significant differences between any of the strains were found in the graphs shown in C-E with p > 0.05 for all comparisons. (TIF) [file pgen.1010938.s005.tif]

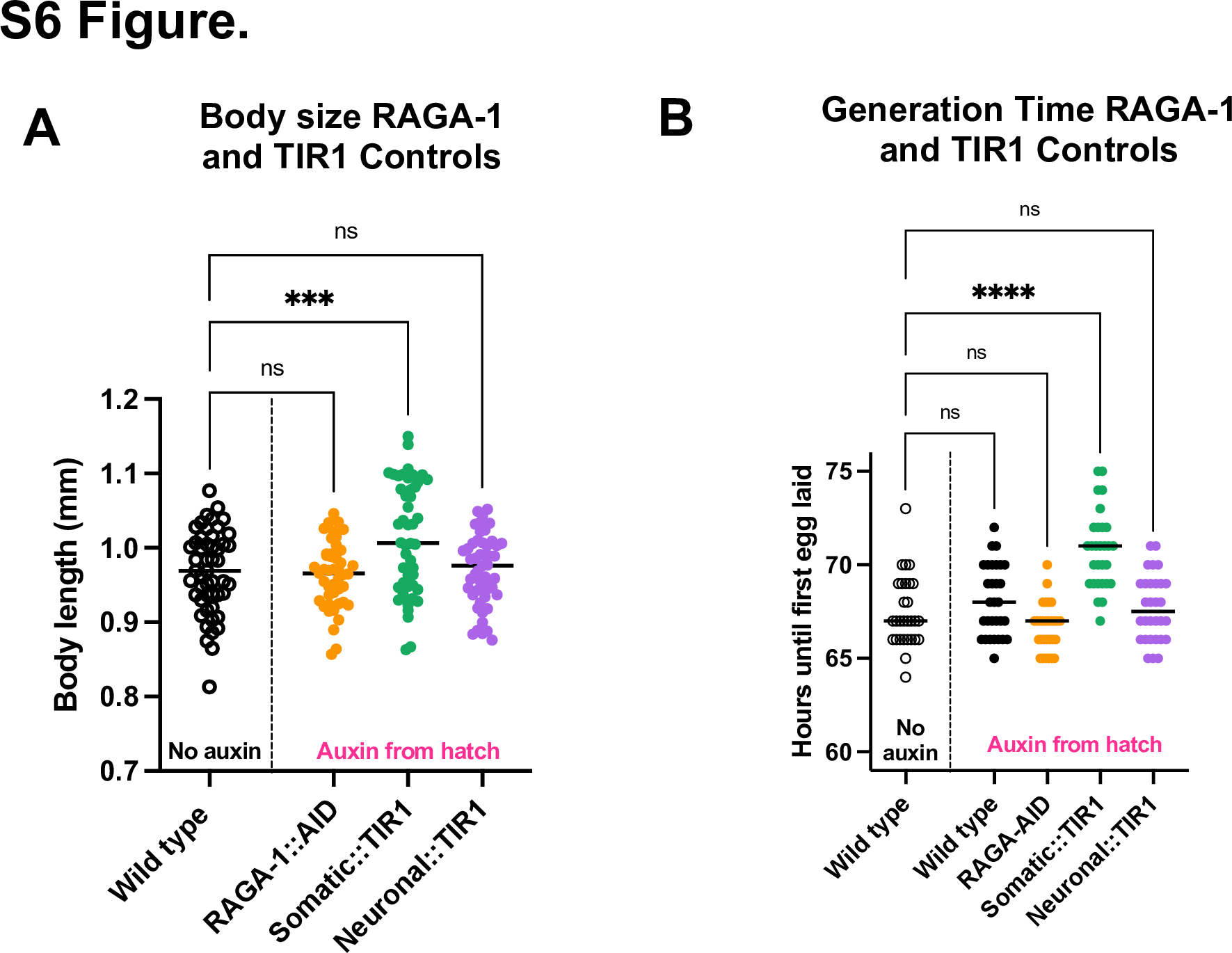

Supplement: S6 Fig — (A) Body size data for RAGA-1::AID control strains. Auxin treatment doesn’t affect body size of RAGA-1::AID or Neuronal::TIR1 animals but Somatic::TIR1 have a mild increase in mean size of 0.045 mm. (B) Generation time for RAGA-1::AID and TIR1 control strains. Auxin treatment doesn’t affect the generation time of wild type, RAGA-1::AID, or Neuronal::TIR1 animals. Somatic::TIR1 animals treated with auxin have a mild (3.3 hours on average) delay in development. n = 2 replicates with at least 29 individual measurements for each condition. *** indicates p < 0.001, **** indicates p <0.0001. (TIF) [file pgen.1010938.s006.tif]

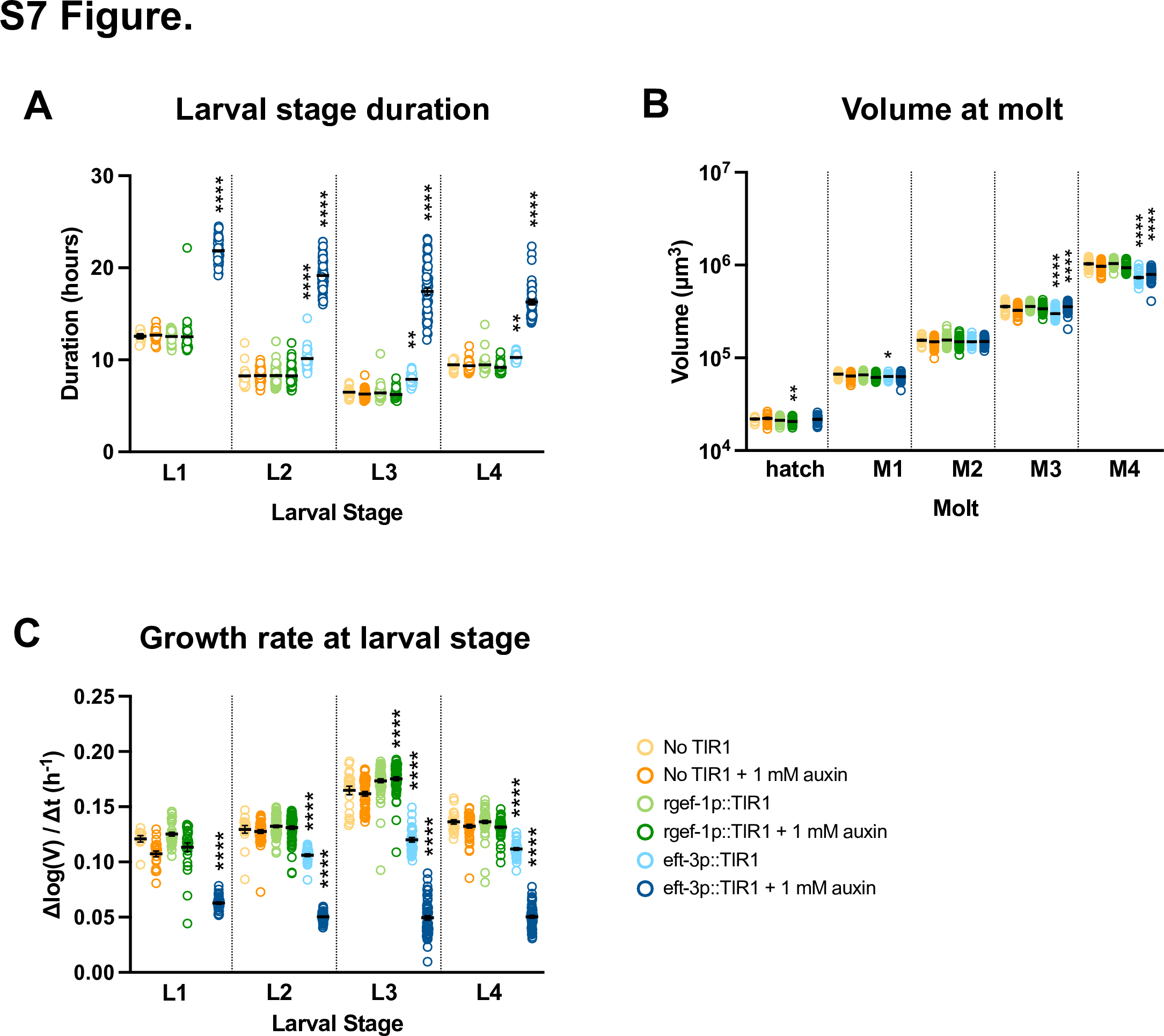

Supplement: S7 Fig — Growth of the RAGA-1::AID strain was measured with and without neuronal (rgef-1p) and somatic (eft-3p) expression of TIR1 in the presence or absence of 1 mM auxin. Individual animals housed in microchambers were imaged to measure (A) time to reach each larval stage, (B) volume at molt, and (C) average growth rate per larval stage. Animals with neuronal RAGA-1 depletion have no impairments in growth measurements throughout the developmental stages. In contrast, somatic depletion of RAGA-1 results in slower progression throughout the larval stages and a slower growth rate. Even in the absence of auxin, the strain expressing TIR1 in the soma has mild growth defects, likely due to auxin-independent degradation of AID-tagged RAGA-1. Data were analyzed by a one-way ANOVA followed by multiple comparisons. All control conditions were analyzed relative to the “No TIR1” group and all conditions on 1mM auxin were analyzed relative to the “No TIR1 + 1 mM auxin” group. Results are not significant (p>0.05) unless otherwise indicated by an asterisk. * indicates p<0.05, ** indicates p <0.01, *** indicates p < 0.001, **** indicates p <0.0001. Each data point represents an individual animal and error bars are plotted at mean ± SEM. Data unavailable for eft-3p::TIR1 strain at hatch and L1 due to technical difficulties. (TIF) [file pgen.1010938.s007.tif]

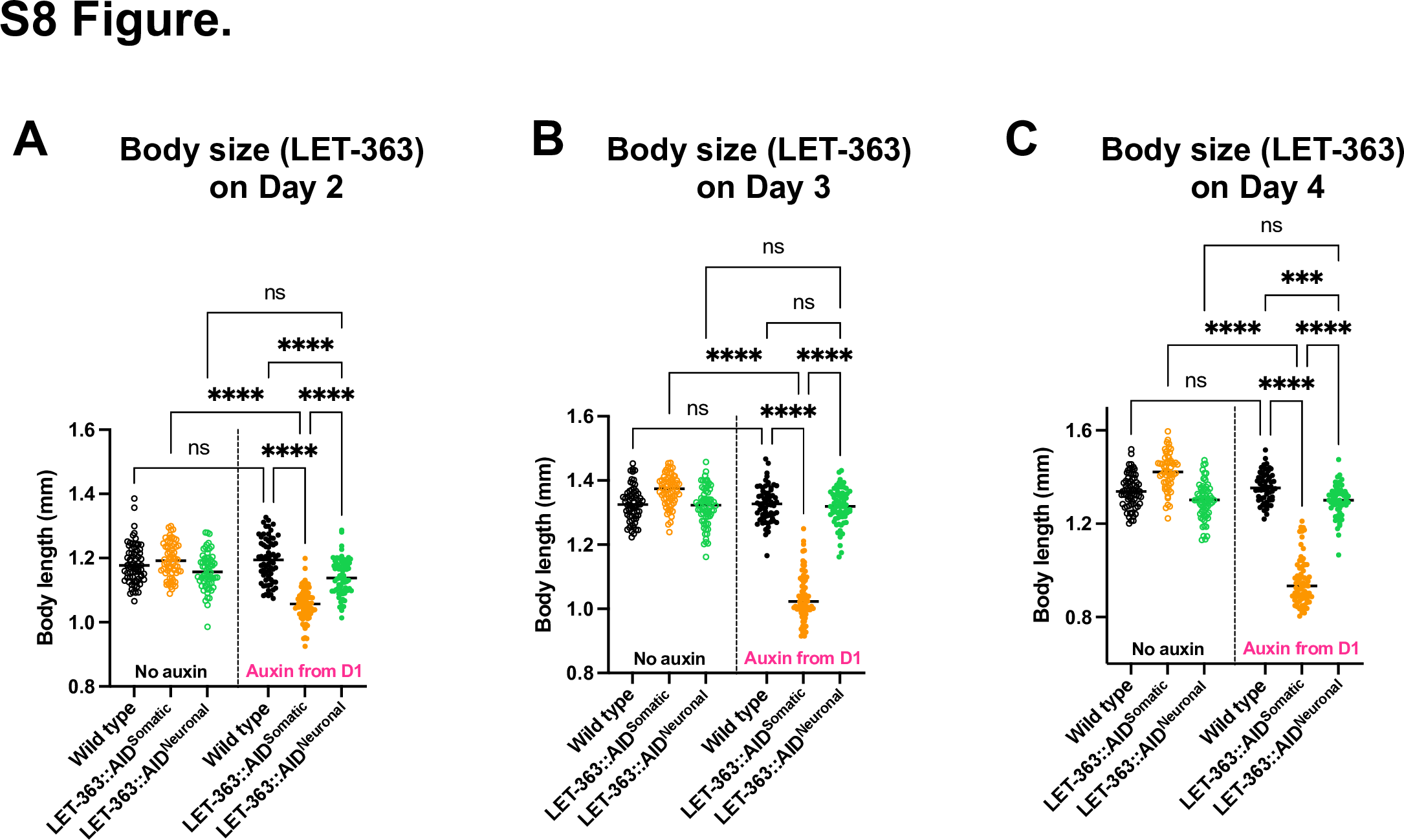

Supplement: S8 Fig — (A-C) These are the same data as in Fig 4C but broken up by day for statistical analysis. Animals were treated with auxin beginning on day 1 of adulthood and body length was measured 24 (A), 48 (B), and 72 (C) hours later. Auxin treatment doesn’t affect body size in wild type animals but results in a significant decrease in body size of LET-363::AIDSomatic animals on all days measured. Auxin treatment of LET-363::AIDNeuronal animals results in a minor (~4%) body size reduction on days 2 (A) and 4 (C) as compared to auxin-treated wild type animals. n = 3 independent replicates with a total of at least 58 individual worms measured for each condition. Each points represents an individual animal (animals from all 3 replicates are combined here) and the black line indicates the mean across all 3 replicates. ns indicates p>0.05, *** indicates p < 0.001, and **** indicates p <0.0001. (TIF) [file pgen.1010938.s008.tif]
