# Supplementary material for: Neuronal mTORC1 inhibition promotes longevity without suppressing anabolic growth and reproduction in C. elegans
Source: PLoS Genet. 2023 Sep 18;19(9):e1010938. doi: 10.1371/journal.pgen.1010938 (PMC10538657; doi:10.1371/journal.pgen.1010938)
Supplement: Attachment — Submitted filename: Response to Reviewers.docx [file pgen.1010938.s012.docx]

Stefan Taubert, PhD
Guest Editor
PLOS Genetics

Gregory Copenhaver
Editor-in-Chief
PLOS Genetics

Dear Editorial Team

We are very pleased to submit a revised version of our manuscript titled ‘Neuronal mTORC1 inhibition promotes longevity without suppressing anabolic growth and reproduction in C. elegans’ for your consideration for publication in PLOS Genetics.

As discussed, we have carried out additional experimentation to fully address the selected reviewer’s comments sent to us by the editorial team. Key new data include:

- Westerns showing that the TORC1 pathway is suppressed via RAGA-1 degradation
- Triplicate repeats testing the requirement of PHA-4 for lifespan extension via neuronal degradation of RAGA-1
- Additional controls for the data generated by our collaborators the Towbin lab
- Imaging analysis to further characterize the AID system.

In addition to new data, we have edited the manuscript and added discussion in response to the reviewers. We have responded to all the points the editorial team highlighted as critical for publication at the top of this rebuttal (our response in blue). In addition, we have made point by point responses to each comment of all 3 reviewers.

We thank all those involved for their service and feel that in response to their input our new manuscript is substantially improved and appropriate for publication in PLOS Genetics

Best

William Mair (on behalf of all authors)

Response to Review:

As you can see in the reviews, all 3 reviewers thought the manuscript was of interest and generally well designed and rigorously analyzed. However, several important points must be addressed, either experimentally and/or in writing:

1. Consider the possibility (experimentally or at least in discussion) that, rather than tissue specificity, allele strength difference may cause the observed phenotypes, as requested by reviewer #1;

We thank the reviewers for this comment and agree that this is an important point to explore. The new data we have added on phospho-RSKS-1 levels following either somatic or neuronal degradation of RAGA-1 addresses this point by showing that, as expected, whole-body mTORC1 activity is significantly decreased following somatic AID of RAGA-1 but is not changed by neuronal AID of RAGA-1. We further elaborated on the idea of allele strength in the discussion and explore this as a possible explanation for phenotypes observed in this manuscript.

2. Reframe the TOR-centric view to a more RAGA-1-centric view as per reviewer #2, to better align with the fact that experiments were focused on the latter;

We thank the reviewer for this suggestion, and we have edited the text to more accurately represent our results as RAGA-1-centric. We have changed many mentions of “mTORC1” to “RAGA-1” throughout the text, especially in the Results section. We have chosen to leave some mentions of the mTORC1 pathway throughout the text, particularly in the Introduction and Discussion, as we believe our data on neuronal LET-363 degradation and our new data showing that RAGA-1 knockdown alters phosphorylation of the mTORC1 target RSKS-1 makes our results somewhat generalizable to the mTORC1 pathway.

3. Ascertain experimentally whether TOR is actually inhibited in the AID strains (RSKS-1 blots), as requested by reviewer #2;

We thank the reviewers for this suggestion and addressed it by performing Western blots for phosphorylation of the mTORC1 target RSKS-1 (putative ortholog of mammalian S6K1), which is known to be decreased with mTORC1 inhibition. We assessed levels of phosphorylated RSKS-1 following neuronal AID of RAGA-1, somatic AID of RAGA-1, and adult-onset somatic AID of LET-363. We observed that somatic AID of RAGA-1 significantly reduces RSKS-1 phosphorylation while neuronal AID of RAGA-1, a much more cell-selective intervention, doesn’t significantly alter RSKS-1 phosphorylation measured from whole animal lysates. Additionally, two days of AID of LET-363 in somatic tissues of adult animals significantly decreased RSKS-1 phosphorylation. Altogether these experiments that whole-body degradation of both RAGA-1 and LET-363 result in reduced RSKS-1 phosphorylation, consistent with mTORC1 inhibition, as expected.

4. Perform some mechanistic follow-up on at least pha-4 in raga-1::AID, and ideally also in let-363::AID, as requested by reviewer #2;

Thank you for this suggestion. We tested whether *pha-4* was required for neuronal RAGA-1 longevity, as that model is the one we focus on the most in the paper and *pha-4* was identified following RNA-Seq on the RAGA-1::AID strains. We found that AID of RAGA-1 in the neurons was still able to extend the lifespan of animals on *pha-4* RNAi, suggesting that *pha-4* is not required for this model of longevity. This work mirrors what has been shown for RAGA-1 mutants (PMID: 20523893).

5. Perform additional control experiments, compare with additional relevant strains, and provide information on experimental details on AID strains, promoters used, and auxin treatments, please see reviewer #2's comments for details;

We have added clarification in the text and methods about experimental details and controls.

6. Provide all data underlying the manuscript, specifically: add a life span data table, as is common practice in the field; this should contain all detailed data on all experimental repeats performed including those presented in the paper in figures as well as replicates shown in supplements or not shown; and add some tables on key gene expression data as supplementary Tables, not just via upload to GEO (this must also be completed).

We thank the reviewers for bringing these points to our attention. We apologize for forgetting to attach the lifespan table in the original submission and have a complete lifespan table, including the new *pha-4* lifespan data, which are now included with the resubmission. We have created an Excel file containing the genes that are either up- or down-regulated following neuronal or somatic RAGA-1 degradation. These tables contain the gene names and expression levels which should be easy for readers to browse and will be submitted as Supplementary Table 2. We have also uploaded the RNA sequencing data set to GEO and it has been assigned the following accession number: GSE237060.

7. The reviewers have proposed several additional experiments that would be interesting but are not considered essential, such as investigation of skn-1, of tissue specific gene regulation effects via GFP reporters, and of AMPK's role in this pathway; if such experiments are available this would further strengthen the paper.

We thank the reviewers for their thoughtful suggestions and agree that many of these experiments would be interesting and build upon our findings. As suggested we added new data on PHA-4. We do not currently have any of these experiments available but look forward to investigating some of these questions as we continue to study neuronal control of aging by the mTORC1 pathway.

Reviewer's Responses to Questions

**Comments to the Authors:
Please note here if the review is uploaded as an attachment.**

**Reviewer #1**: In this manuscript entitled “Neuronal mTORC1 inhibition promotes longevity without suppressing anabolic growth and reproduction in C. elegans”, the authors functionally dissected the effects of neuron-specific inhibition of mTORC1 on the longevity, growth, and reproduction of C. elegans. The authors found that neuron-specific degradation of RAGA-1, an upstream regulator of mTORC1, using AID extended the lifespan of worms. The authors then showed that the neuron-specific depletion of RAGA-1 did not disrupt the growth or reproduction of animals, which was impaired by somatic degradation of RAGA-1. The authors also found that neuronal depletion of LET-363, C. elegans mTOR, promoted the longevity of animals while had no effects on the growth and development of worms. Lastly they analyzed genes whose expression was changed by neuronal and somatic RAGA-1 inhibition using RNA seq, and showed that neuronal depletion of RAGA-1 affects the expression of small number of genes compared with somatic degradation of RAGA-1. Overall, this interesting work demonstrates that specific inhibition of mTOR signaling can promote longevity without defects in growth and reproduction. This study was well designed, the experiments were rigorously done, and the paper was well written. I have only a few major comments, which can further improve the paper.

Major comments
1. One alternative explanation about the key data here is that perhaps neuronal inhibition of mTOR is a proper reduction of function that causes longevity without growth/reproduction defects whereas the somatic inhibition is a strong loss of function that causes severe effects on growth/reproduction and impairs health. In fact, their PCA in Figure 5A indicates typical strong vs. weak allele that affect gene expression differentially. I wonder whether they could test this possibility by using different concentrations of auxin with somatic mTOR signaling depletion strains, and determine whether they could achieve longevity without growth/reproduction defects at some lower concentrations. Or at least they need to add discussion point about this possibility.

We thank the reviewer for this observation. We were not able to test the effects of different auxin concentrations, as we find that even after titrating auxin down to a very low level, it still induces strong degradation of AID-tagged proteins. However, we agree that this is an interesting possibility and have expanded on this in the Discussion section.

2. Related to the comment 1, I wonder whether the authors can analyze their RNA seq data to test whether there are overall qualitative or quantitative differences in gene expression changes by neuronal vs. somatic RAGA-1 depletion. I think there must be both aspects, but the current Figure 5 and description in the text do not address the point.

We thank the reviewer for raising this point. To investigate this, we have performed additional analysis of the RNA sequencing data. We plotted the fold change of genes that were regulated in the same direction (up in both data sets or down in both data sets) following somatic and neuronal AID of RAGA-1. We found 103 overlapping upregulated genes and 21 overlapping downregulated genes. When plotted against each other, you can see that for the upregulated genes, many genes are similarly upregulated following somatic and neuronal AID of RAGA-1. Indeed, some genes are more highly upregulated following somatic AID of RAGA-1, but others are more highly upregulated following neuronal AID of RAGA-1. For the downregulated genes, all of the genes have a slightly lower fold change following somatic AID of RAGA-1, indicating that their expression is downregulated more by somatic than neuronal AID of RAGA-1. We have not added these data to the paper however, as we feel detail comparison of the effects that somatic and neuronal RAGA degradation requires tissue specific ribosome profiling, for which we have recently generated a new tool kit and are actively pursuing in ongoing work.

Minor comments

1. On page 9, please add proper references for auxin-independent degradation of AID-tagged proteins.

We have added this reference.

2. In figure 3 and figure 4, please add statistical significance among auxin-treated animals. In addition, please doublecheck the panel names of the figure 3 legends. There should be no panel D.

Thank you for pointing this out. We have reviewed the figure legends and double-checked the labeling and references to the panels. Additionally, we have added statistical comparisons between the auxin-treated animals.

3. In figure 3 – supplement 1, please add proper title for the figure in the legends.

Thank you for noting this. We have corrected the legends.

4. On pages 14 and 26, please add a reference for “Stojanovski et al.”

We apologize for missing references and have corrected this. Thank you.

**Reviewer #2**: In this manuscript, the authors investigate the tissue-specific role of mTORC1 pathway inhibition in extending lifespan. They use the AID degron system to degrade RAGA-1 or LET-363/mTOR in C. elegans neurons only, and find that lifespan extension is achieved without causing other physiological defects associated with whole-body mTOR inhibition such as reduced body size and progeny production. This suggests that lifespan benefits can be uncoupled from the ‘side effects’ of mTOR inhibition. The authors present data using a novel tool for neuron-specific mTOR pathway inhibition, and present a solid manuscript that illustrates the importance of tissue-specific analyses of genes involved in lifespan analyses, but may represent incremental novelty besides the development and validation of new tools.
Major comments:

**mTOR inhibition**
1. The bulk of the data was produced using the RAGA-1::AID strains, not the LET-363::AID strains. Several changes must be made to the claims in the manuscript to downplay the role of “LET-363/mTOR inhibition” and instead clarify that the findings are a result of RAGA-1 degradation. Unless a full panel of tests is performed in the LET-363::AID strains as was done with the RAGA-1::AID strains, the data in Fig 4 showing the effects of auxin-induced LET-363/mTOR degradation should be used to support the RAGA-1 findings. To this end, the direct role of mTOR inhibition should be downplayed (lines 286-300).

We appreciate this comment and have addressed the reviewer’s concerns. We agree that the LET-363 data is mostly supporting the RAGA-1 data. We have changed many instances of “mTORC1” to “RAGA-1” in order to refocus the manuscript. Moreover, we performed a Western blot for RSKS-1 phosphorylation and found that AID of RAGA-1 does indeed decrease phosphorylation of RSKS-1, a downstream target of mTORC1. Thus, we believe that some reference to the mTORC1 pathway in general is appropriate, but we have greatly reduced the mention of mTORC1 and have refocused the manuscript on RAGA-1.

2. Throughout the manuscript, the authors use the term “mTOR inhibition” to refer to AID degradation of either RAGA-1 or LET-363. This term is inappropriate if mTOR inhibition is not actually shown and could be challenging to be shown tissue-specifically, which would represent a significant advance if the authors could show that. Nevertheless, the authors need to test if neuronal mTOR inhibition can be shown by assessing phosphorylation of mTOR substrate RSKS1/S6K by Western blot in control and auxin-treated worms.

Thank you for this comment. As written above, we have now assessed mTOR inhibition by RSKS-1 Western blot in control and auxin-treated worms. We observed that somatic AID of RAGA-1 significantly reduces RSKS-1 phosphorylation while neuronal AID of RAGA-1, a much more acute intervention, doesn’t significantly alter RSKS-1 phosphorylation measured from whole animals. Additionally, two days of AID of LET-363 in somatic tissues of adult animals significantly decreased RSKS-1 phosphorylation. Thus, AID of RAGA-1 and LET-363 are indeed inhibiting the mTORC1 pathway. Nevertheless, we have changed many instances of “mTOR inhibition” to “RAGA-1 degradation” to more accurately represent our studies.

**Differential expression analyses**
1. Fig 5 contains some interesting differential expression data; however, there is no follow-up to these findings. The authors could considerably strengthen their work by showing that some of the upregulated genes are required for lifespan extension. Since pha-4 is required for lifespan extension by let-363 RNAi, and identified as an upregulated upon neuronal RAGA-1 inhibiton, the authors must test whether pha-4 is required for lifespan extension upon neuronal RAGA-1::AID degradation (and ideally let-363 AID). This could also be done with skn-1 and/or its target genes and chromatin remodeling or autophagy genes.

Thank you for this suggestion, we agree that there are many interesting follow-up studies to be performed on the RNA sequencing data that will expand our understanding of the mechanisms of neuronal RAGA-1 longevity and look forward to testing some of these hits in future studies. For the purposes of this manuscript, we have now tested whether *pha-4* is required for lifespan extension upon neuronal RAGA-1::AID degradation. We found that when *C. elegans* were placed on *pha-4* RNAi, neuronal RAGA-1 degradation was still able to extend their lifespan, suggesting that *pha-4* is not required for this longevity paradigm. This result narrows down our pool of possible factors causal to lifespan extension and we look forward to testing others in the future.

2. In lines 355-359, the authors reference their differential expression data shown in Fig 5, proposing that “neuronal mTORC1 is able to cell nonautonomously regulate gene expression in peripheral tissues (lines 358-359). This claim would be much stronger if at least one example of these cell non-autonomous effects was shown. For instance, gst-4 and/or gcs-1 are targets of SKN-1 expressed in the neurons and muscle, and intestine, respectively. Transcriptional reporters could be used as a readout for cell non-autonomous SKN-1-mediated stress response upon neuronal RAGA-1 degradation.

We thank the reviewer for this comment. We see specific examples from our Seq data of changes in expression of non-neuronal genes when we degrade RAGA-1 in the nervous system. In the text we now state “we find that neuronal RAGA-1 degradation induces differential expression of non-neuronal genes - such as plin-1, gnsl-1, myo-2, and ges-1 – suggesting that neuronal mTORC1 is able to cell nonautonomously regulate gene expression in peripheral tissues.”

Although we agree that a fluorescent reporter is another method to show this same effect, we believe they are less direct read outs than methods that look at levels of endogenous RNAs. We feel detail comparison of the cell nonautonomous effects of neuronal RAGA degradation requires tissue specific ribosome profiling, for which we have recently generated a new tool kit and are actively pursuing in ongoing work, but that we feel is beyond the scope of this manuscript.

**Neuronal RAGA-1 and LET-363 inhibition**
1. The authors show that RAGA-1 is reduced in somatic tissues, but not in neuronal tissues. The authors need to show that RAGA-1 and ideally LET-363 is reduced specifically in neur using their RAGA-1::AIDNeuronal strain. The authors are also two different RAGA-1::AIDneuronal strains, rab-3::TIR1 and rgef-1::TIR1. The authors need to explain the use of two different promoters and compare and contrast the knockdown efficiency. In Fig 1 (and throughout the manuscript), no images are shown of neuronal RAGA-1::AID::EmGFP expression. Images of the head and other body regions of this strain are needed to assess the expression pattern.

We apologise if there is some confusion here: ‘Somatic degradation’ includes degradation in neurons as it included all somatic cells. We see complete loss of GFP signal in the head of the worm in the Somatic TIR1 strain, so there is no reason to think the neurons would be refractory to the AID system.It has been technically challenging to image neuron-specific degradation of RAGA-1::GFP due to the persistence of GFP signal in other tissues in the head such as the hypodermis and pharynx. The strain we are using to express TIR1 has been characterized to have robust neuron-specific expression and were generated using single copy knock in with neuronal 3’ UTRs, so their expression is robust and invariable (PMID: 31064766). We have included new confocal images showing baseline RAGA-1::AID::EmGFP throughout whole worm (Figure 1C). The second neuronal promoter, RGEF-1, was used in a second site by our collaborator to add a second method of showing the same effect, as it indeed did.

**Lifespan extension by AID of RAGA-1 in soma and neurons**

1. In lines 191-193, authors claim that the lifespan extension seen in RAGA-1::AIDSomatic animals even without auxin treatment may be due to “a low level of RAGA-1 degradation, as mild auxin-independent degradation of AID-tagged proteins has been previously reported”. First, this statement should be referenced. Moreover, this could easily be tested by imaging and comparing fluorescence intensity in stains 1 and 1x3 (as shown in figure 1A) +/- auxin treatment.

Thank you for these observations and suggestions. We apologize for missing references and have corrected this citation as well as references throughout the text. Additionally, we performed imaging to quantify the degree of auxin-independent degradation that occurs in our strains upon the introduction of Somatic::TIR1. As suspected, the introduction of Somatic::TIR1 into the RAGA-1::AID::EmGFP strains results in a significant decrease in GFP expression. Upon auxin treatment, the GFP expression is further diminished down to the levels of GFP autofluorescence found in wild type animals. In contrast, the auxin-independent degradation is not observed in the Neuronal::TIR1 line. This could be due to the fact that the neurons make up a small volume of the animal and are masked by the persistence of GFP signal in surrounding tissues such as the pharynx and the hypodermis.

2. It is not clear when RAGA-1::AID Somatic animals are placed on auxin in this experiment, This informatin need s to be included in line 194 and the figure legend. Moreover the authors shold include a d1 auxin control into Figure 2A, if this is not the d1 experiment.

Thank you for this comment. Auxin onset will be clarified in text and legend. Although we do not have a D1 auxin control in this RAGA-1 lifespan, the LET-363 lifespans have a condition in which WT animals are placed onto auxin beginning at D1 and we observe no difference in lifespan.

3. In Fig 2A, it would be helpful to include raga-1 mutant lifespans +/- auxin as a control. Since there are several raga-1 mutants, as discussed by the authors, perhaps the two with the most and least change in lifespan should be included.

We thank the reviewer for this point but given the short time frame for resubmission this additional negative control was not possible.

**Lifespan data**
1. The lifespan table is not included in the manuscript. This is a critical set of data that will have to be assessed, especially since claims throughout the text of the manuscript refer to it.

We have now included this table as Supplementary Table 1.

2. For all lifespan graphs, day of onset of auxin treatment should be clearly indicated in the figure and legend.

We have now double checked all legends and corrected where necessary

**Inhibiting mTORC1 in the neurons uncouples aging from anabolic trade-offs.**
1. The authors are performing several experiments to show that development, growth, and reproduction are unaffected upon neuronal inhibition of RAGA-1. Fig 3 would benefit from raga-1 mutant comparisons as a positive control. In Fig 3 S1, no-auxin controls are missing for the three strains that are shown as treated with ‘auxin from hatch’. These controls are required to show whether the observed differences in development are due to auxin treatment and not construct expression.

We thank the reviewer for this comment. Although we can see how adding the *raga-1* mutant would add more information, we believe the more pertinent positive control is simply using the somatic degradation of RAGA-1. We believe it is more relevant since it is knocking down RAGA-1 using the same system as how we knock it down in neurons, and most of our paper is focused on analyzing differences between somatic and neuronal degradation, not comparing to the loss-of-function mutant.

To the second point, although the no auxin controls are not included, we believe that we have other strains present within our experiments to control for construct expression. The hypothesis that observed differences in development are due to construct expression rather than auxin treatment is refuted by the fact that the animals expressing raga-1::AID and TIR1 have no developmental differences in the “no auxin” conditions. We will make this more clear in the text.

2. Figure 3 supplement 2: Suggestion to include RAGA-1::AIDSomatic strain in Fig 3 S2 subfigures, as this is an important comparison to test whether the differences that are statistically significant are meaningful relative to whole-body degradation of RAGA-1. Alternatively, this figure could be deleted, as it is not crucial to the main findings and performed with a different TIR1 expressing strain.

We have now included new data from the Towbin lab that has Somatic strain included

**Minor comments:**
- Line18 (abstract): change “specifically to C. elegans” to “specifically in C. elegans”

We have changed the sentence for clarity to: “Here, we utilize the auxin-inducible degradation (AID) system to restrict mTORC1 inhibition to *C. elegans* neurons”

- Line 39-40 (author summary): clarify whether “unwanted side effects such as impaired growth, development and reproduction” applies to multiple model organisms or only C. elegans

We have clarified that the “unwanted side effects” apply to multiple model organisms.

- Line 81-83: The authors should define Y allocation theory

Thank you for this suggestion. We have now defined Y allocation theory.

- Line 94-95: Define or clarify Drosophila terms used in the introduction and discussion, such as “fat-specific” 9GAL4-UAS”, “fat-body” (line 401-402).

Thank you for pointing this out. We have clarified its use for tissue-specific expression.

- Line 153: Several degron systems have been developed for use in C. elegans, including mini-AID (mAID) and AID2. The authors should clarify which system was used.

Thank you for this observation. We are using the original AID system, not mAID or AID2, that is used in the Zhang et al. 2015 paper that we cite in the results and methods section. In addition, in the Methods section, In the methods section, we have added a section where we mention the plasmids from which we amplified the degron tag and the TIR1, which should clear up any confusion.

- Line 169: “Fig 3A-D” does not correspond to the actual figures shown. Figure legend also does not match the figure.

Thank you for noting this. We have fixed the figure legends and references within the text.

- Line 188: change “all somatic tissue” to “all somatic tissues”

We have corrected this in the manuscript.

- Line 217-218: missing reference for requirement of splicing factor SFA-1.

We have corrected the reference.

- Line 257: change “moult” to “molt”.

Thank you for noting this, we have corrected the spelling.

- Line 259: The Stojanovski et al., reference is not in the reference list.

We have added the reference.

- Line 276: suggest to change “will be true” to “could be true”.

We have changed the text to “could be true”.

- Line 297: remove “pro-aging phenotypes” since mTOR-inhibition-induced sickness is not equivalent to aging.

We have changed “pro-aging” to “deleterious.”

- Line 305: change “72 hours post auxin treatement” to “72 hours post onset of auxin treatment”, since auxin treatment is not stopped.

We have changed the text, thank you for this suggestion.

- Line 371-373: missing reference for “our previous finding … whole body mTORC1 inhibition”.

We have added the reference.

- Line 397: suggestion to change “sufficient for longevity” to “sufficient to promote longevity”.

We have changed the text to “sufficient to promote longevity”.

- Line 787-788: revise syntax.

The figure caption has been revised for clarity.

- Line 809-812: the significance of the asterisks for statistical comparisons should be stated, as done in line 854.

Thank you for this suggestion. We have added an explanation of the asterisks in the Statistical Analysis section of the Methods.

- Suggestion to combine Fig 2 S2 A and B into one graph to be able to compare RAGA-1::AID conditions to PR811.

Thank you for this suggestion. We agree that this would be the easiest to interpret but we have decided to leave the graphs separate since the experiments were performed on separate days. However, the scales on both graphs are the same and the osm-6 mutant consistently performs significantly worse than the RAGA-1::AID strains.

- In Fig 2 S2B, statistical comparison between wild type and RAGA-1::AID within treatments is more important than between treatments.

Thank you for noting this, we have added the statistical comparisons within treatments.

- Suggestion to use linear regression or other function as a statistical model for Fig 2 S2D, as there seems to be some difference between wild type +/- auxin.

We performed linear regression of wild type +/- auxin in GraphPad Prism and then tested whether the slopes or intercepts were significantly different. We found that neither the slopes, p = 0.95, nor the intercepts, p = 0.8103, varied significantly. We can see that the lines might appear to have some difference on the graph, but please note the error bars for the lines overlap a lot, likely contributing to the nonsignificant statistical outcome.

- Statistical test used is not indicated in the figure legend for Fig 2 S2A.

We have added information on the statistical tests used.

- Suggestion to combine Fig 4 C-E into one graph with ‘day of adulthood’ on the x-axis and ‘mean body size +/- error) on the y axis to better represent the differences in trends between somatic LET-363::AID and the other two strains.

Thank you for this suggestion, we agree that it will increase clarity. We have combined the body size data into one graph to make it easier to see all of the data. However, we felt this left the figure too crowded to add statistics directly on top of it, so we have added the original graphs to a supplemental figure (Figure 4 – supplement 1) where all the statistical comparisons can be seen.

- A comparison between the roles of RAGA-1 and LET-363 in the context of the mTOR pathway, lifespan extension and other phenotypes could strengthen the Discussion section. Otherwise, this section could be shortened significantly.

Thank you for this suggestion. We have edited the Discussion to shorten it but we believe discussing topics such as the benefits of the AID system and the broader context of what is known about how neurons regulate longevity will be beneficial for readers. We mention that LET-363 is a component of both mTOR complex 1 and complex 2, which could explain some of its difference with phenotypes observed from RAGA-1. Additionally, we mention information about various *raga-1* mutant alleles and the effects of RagA knockout in mammals. We hope this information provides readers with some insight into the context of the mTOR pathway.

**Reviewer #3**: This is an interesting and well conducted study by the Mair group together with the Towbin lab. The question that is addressed is an important one and the paper is methodologically sound and appears well conducted. I am not an expert on the specific approach (Auxin-inducible degradation), but the method appears elegant and quite powerful. Using this approach, the authors address the question whether the fitness tradeoffs that are typically associated with lifespan effects (benefits) seen with reduction of mTOR signaling are obligatory. Such tradeoffs are commonly assumed to be mechanistically directly linked to mTOR-related benefits, suggesting that these two effects should be impossible to uncouple. The authors provide strong evidence that this is not in fact the case, at least in C. elegans.
The AUX system requires relatively high concentrations of auxin I was initially somewhat concerned that the observed effects might be impacted by AUX off-target effects. However, the authors have carried out several control experiments and I think the data is quite conclusive. The lifespan effects of neuronal AID RAGA-1 are also quite similar to those previously reported with adult RNAi, again supporting the interpretation.

Minor comments:

The observation that neuronal only degradation of RAGA-1 extends lifespan yet only affects a limited number of target genes (compared to somatic RAGA-1 - as judged by RNAseq) is very interesting. The authors state that data will be made available by upload to GEO – I would suggest (in addition) to provide the DEG statistics in csv or xls format as part of supplementary data.

Thank you for this suggestion, we agree this would be a valuable resource. We have now created Excel files that will be included as Supplementary Table 2 that list the gene name and expression of the significantly upregulated and downregulated genes following either neuronal or somatic degradation of RAGA-1.

Previous work has reported that neuronal AMPK is required for lifespan extension by mTOR inhibition in C. elegans. It would interesting to determine if neuronal AMPK is similarly required for lifespan extension by RAGA-1 degradation in neurons using the AUX system.
Given these previous data regarding interactions between neuronal AMPK and mTOR-mediated lifespan determination, I would suggest that this aspect should be at the very least discussed in light of the new results presented here.

We thank the reviewer for this suggestion. Although we have not performed these experiments, we have added a section in the Discussion which encompasses the previous findings on AMPK.

**Have all data underlying the figures and results presented in the manuscript been provided?**
Large-scale datasets should be made available via a public repository as described in the *PLOS Genetics* [data availability policy](http://journals.plos.org/plosgenetics/s/data-availability), and numerical data that underlies graphs or summary statistics should be provided in spreadsheet form as supporting information.

Reviewer #1: Yes

Reviewer #2: **No:** No Lifespan table provided to reviewers

Reviewer #3: Yes

PLOS authors have the option to publish the peer review history of their article ([what does this mean?](https://journals.plos.org/plosgenetics/s/editorial-and-peer-review-process#loc-peer-review-history)). If published, this will include your full peer review and any attached files.


If you choose “no”, your identity will remain anonymous but your review may still be made public.


**Do you want your identity to be public for this peer review?** For information about this choice, including consent withdrawal, please see our [Privacy Policy](https://www.plos.org/privacy-policy).

Reviewer #1: **Yes:** SEUNG-JAE V. LEE

Reviewer #2: No

Reviewer #3: No
